# Opportunities and Challenges of Bacterial Glycosylation for the Development of Novel Antibacterial Strategies

**DOI:** 10.3389/fmicb.2021.745702

**Published:** 2021-09-24

**Authors:** Liubov Yakovlieva, Julius A. Fülleborn, Marthe T. C. Walvoort

**Affiliations:** Faculty of Science and Engineering, Stratingh Institute for Chemistry, University of Groningen, Groningen, Netherlands

**Keywords:** pathogenic bacteria, glycosylation, antivirulence, antibacterial strategies, metabolic oligosaccharide engineering

## Abstract

Glycosylation is a ubiquitous process that is universally conserved in nature. The various products of glycosylation, such as polysaccharides, glycoproteins, and glycolipids, perform a myriad of intra- and extracellular functions. The multitude of roles performed by these molecules is reflected in the significant diversity of glycan structures and linkages found in eukaryotes and prokaryotes. Importantly, glycosylation is highly relevant for the virulence of many bacterial pathogens. Various surface-associated glycoconjugates have been identified in bacteria that promote infectious behavior and survival in the host through motility, adhesion, molecular mimicry, and immune system manipulation. Interestingly, bacterial glycosylation systems that produce these virulence factors frequently feature rare monosaccharides and unusual glycosylation mechanisms. Owing to their marked difference from human glycosylation, bacterial glycosylation systems constitute promising antibacterial targets. With the rise of antibiotic resistance and depletion of the antibiotic pipeline, novel drug targets are urgently needed. Bacteria-specific glycosylation systems are especially promising for antivirulence therapies that do not eliminate a bacterial population, but rather alleviate its pathogenesis. In this review, we describe a selection of unique glycosylation systems in bacterial pathogens and their role in bacterial homeostasis and infection, with a focus on virulence factors. In addition, recent advances to inhibit the enzymes involved in these glycosylation systems and target the bacterial glycan structures directly will be highlighted. Together, this review provides an overview of the current status and promise for the future of using bacterial glycosylation to develop novel antibacterial strategies.

## Introduction

Bacterial pathogens have evolved an extensive arsenal of strategies to persist and thrive in the host. These strategies are referred to as “virulence factors,” and in the process of host infection, they directly or indirectly contribute to enhanced survival of the bacterium (Clatworthy et al., 2007). Interestingly, many of the virulence factors are glycosylation products, in the form of either oligo- and polysaccharides (capsule and LPS) or glycoproteins (pili, flagella, adhesins, autotransporters, and efflux pumps). Additionally, bacterial glycosyltransferases themselves can act as exotoxins, manipulating the host immune response *via* glycosylation of the host proteins.

**Figure 1:**
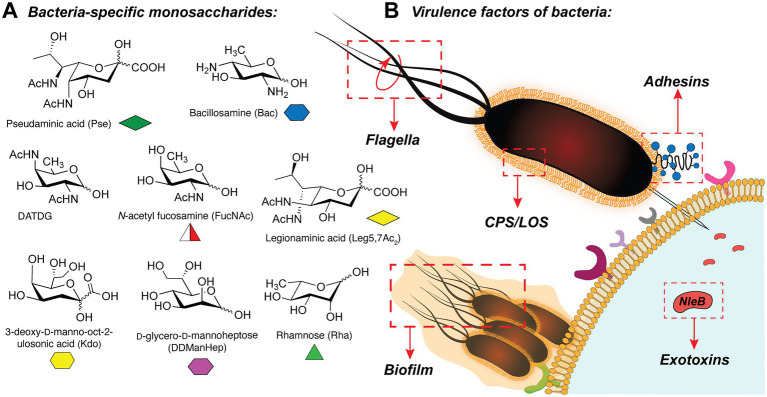
**(A)** Selection of bacteria-specific monosaccharides featured in infectious glycan structures; **(B)** Schematic overview of the bacterial glyco-virulence factors discussed in this review.

In bacteria, the synthesis of glycoconjugates takes place in the series of glycosylation reactions, in which carbohydrates are polymerized or attached to the proteins or lipids, by the action of glycosyltransferase enzymes (GTs). Interestingly, bacterial glycans frequently contain unique monosaccharides such as pseudaminic acid (Pse; Schirm et al., 2003), bacillosamine (Bac; Morrison and Imperiali, 2014), 2,4-diacetamido-2,4,6-trideoxygalactose (DATDG; Hartley et al., 2011), *N*-acetylfucosamine (FucNAc; Horzempa et al., 2008), legionaminic acid (Leg; Morrison and Imperiali, 2014), 3-deoxy-d-manno-octulosonic acid (Kdo; Lodowska et al., 2013), rhamnose (Rha; Mistou et al., 2016), and others (Chatterjee and Chaudhuri, 2003; Meeks et al., 2004; Tytgat and Lebeer, 2014; Figure 1A). These carbohydrates are presented in the glycan structures of several clinically relevant pathogens (for instance, *Helicobacter pylori, Neisseria meningitidis, Pseudomonas aeruginosa, Campylobacter jejuni, Escherichia coli,* among others) and are often important for their virulence (Schirm et al., 2003; Horzempa et al., 2008; Hartley et al., 2011; Hopf et al., 2011; Clark et al., 2016).

Given the importance of glycans as bacterial virulence factors, the biosynthetic machineries that work on these unusual carbohydrates are interesting targets for novel antibacterial therapeutics (Bhat et al., 2019). To date, prominent antibiotics that target bacterial glycans are small-molecule inhibitors of peptidoglycan production (Tra and Dube, 2014). Among those, the best known are broad-spectrum antibiotics such as penicillin (Park and Strominger, 1957) or vancomycin (Perkins, 1969). Although the use of these drugs has met large success in the clinic, significant drawbacks are associated with these therapeutics. Firstly, the misuse of antibiotics has led to a rapid development of multi-resistant bacteria that are now unsusceptible to most antibacterial treatments (World Health Organization, 2014). Secondly, antibiotics do not act strain specifically and thus cause damage to the commensal gut microbiome leading to side effects and further health complications such as infections with opportunistic pathogens like *Clostridium difficile* (Keeney et al., 2014). Therefore, there is a high demand for novel bacteria-specific therapeutics.

Alternative strategies, in which the virulence factors of pathogenic bacteria are therapeutically targeted, have gained more attention over the years (Clatworthy et al., 2007; Dickey et al., 2017). Drugs targeting virulence factors are collectively called antivirulence drugs or pathoblockers (Calvert et al., 2018). Because virulence factors are not essential for the survival of most bacterial pathogens, their inhibition puts little selective pressure on the organisms for the development of resistance (Calvert et al., 2018). Furthermore, many virulence factors are pathogen-specific, and antivirulence drugs hold the promise to act in a strain-specific way and thereby do not exhibit harmful effects of broad-spectrum antibiotics on the gut microbiome (Dickey et al., 2017). Importantly, a multitude of bacterial virulence factors are glycosylation products, including oligo- and polysaccharides, glycoproteins, and glycosyltransferase effector proteins. They feature bacterial species-specific monosaccharides and have unique structures which make them promising candidates for the antivirulence therapies. Although to date no antivirulence drugs are widely used in the clinic, there are already Food and Drug Administration (FDA)-approved antivirulence therapeutics available and many more in the stage of clinical or preclinical development (Dickey et al., 2017). Several experimental approaches have been developed that target bacterial GTs, biosynthetic enzymes of rare bacterial carbohydrates, and metabolic inhibitors of glycosylation (Ménard et al., 2014; El Qaidi et al., 2018; Williams et al., 2020). Together, these methods may provide future directions for the treatment of bacterial infections by targeting the bacterial glycosylation machinery.

In this review, the idea of targeting bacterial glycosylation systems for the development of novel antibacterial therapeutics is explored. Several important classes of bacterial virulence factors are discussed, alongside the strategies developed for their inhibition. Finally, we discuss the potential new glycosylation targets for inhibitors and provide the outlook and future perspectives.

## Part 1: Glycosylation of Bacterial Virulence Factors and Inhibition Strategies

### Motility

#### Flagellar Glycosylation of *C. jejuni* and *H. pylori*

Many pathogenic bacteria rely on motility during different stages of their infection process (Figure 1B). Especially, flagellar motility has been shown to play a critical role in successful infection in many organisms, as it contributes to bacterial movement, adhesion, and biofilm formation. In addition, the glycosylation of flagella is crucial for the proper assembly of flagellar structures and their motility function (Logan, 2006; Merino and Tomás, 2014).

**Figure 2:**
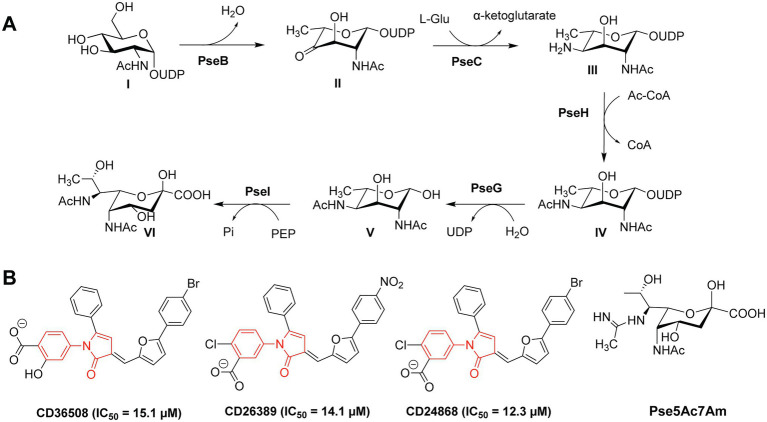
**(A)** Biosynthetic pathway of Pse in *Campylobacter jejuni* and *Helicobacter pylori*; **(B)** Structures of the PseB inhibitors CD36508, CD26389, and CD24868 with their respective IC_50_ values in μM (Ménard et al., 2014).

Flagellar glycans feature diverse structures and often incorporate bacteria-specific monosaccharides. For instance, in the gastric pathogens *H. pylori* and *C. jejuni* the unique bacterial carbohydrates pseudaminic acid (Pse), legionaminic acid (Leg), and derivatives containing acetamidino and methylglycerol moieties are required for the proper assembly of flagella (Ud-Din and Roujeinikova, 2018). Biosynthesis of Pse is a multi-step process that relies on several enzymes (PseB-PseI), as shown in Figure 2A (Ud-Din and Roujeinikova, 2018). *H. pylori* and *C. jejuni* strains expressing non-functional Pse biosynthesis genes show defects in the formation of flagella and are thus non-motile and less virulent (Linton et al., 2000; Goon et al., 2003; Schirm et al., 2003; Guerry et al., 2006; Schoenhofen et al., 2006; Hopf et al., 2011; Javed et al., 2015a). Consequently, the inhibition of the Pse biosynthesis in these bacterial species is a promising antibacterial strategy.

Small-molecule inhibitors of Pse biosynthesis enzymes of *H. pylori* were identified using high-throughput screening (HTS) and virtual screening (VS) approaches in combination with kinetic studies and structure–activity relationship (SAR) analysis (Ménard et al., 2014). Ultimately, three PseB inhibitors were identified with a conserved *N*-phenyl-2-pyrrolidone core featuring different substitution patterns on the phenyl groups (Figure 2B). These three PseB inhibitors exhibited IC_50_ values of ~14μM *in vitro* on purified PseB enzymes. Importantly, the inhibitors were also able to penetrate the bacterial cell wall and inhibit flagellin production in *C. jejuni* in a dose-dependent manner as determined by whole cell ELISA. The relatively low IC_50_ and the ability to cross the bacterial cell wall make these compounds interesting molecules for further development into clinical antibacterial drugs.

In addition to the *O*-linked flagellin glycosylation with unmodified Pse, *C. jejuni* also decorates its flagellin with Pse variants, mainly Pse derivative 7-acetamidino-Pse (Pse5Ac7Am; Figure 2B) in which an acetamido group has been substituted for an acetamidino moiety (Thibault et al., 2001; Schirm et al., 2005; Logan et al., 2009). Interestingly, the phage protein FlaGrab [previously called Gp047 (Sacher et al., 2020)] of the *Campylobacter* phage NCTC 12673 specifically binds to Pse5Ac7Am-modified flagellins of *C. jejuni*, resulting in reduced motility and partially inhibition of cell growth (Javed et al., 2015a,b). The C-terminal flagellin binding domain of FlaGrab has only been used for the detection of *C. jejuni* and *C. coli* so far (Singh et al., 2011, 2012; Javed et al., 2013). While a therapeutic use of FlaGrab against *C. jejuni* infections remains attractive, it has not yet been further explored. Notably, the *C. jejuni* strains 12661 and 12664 show reduced binding by FlaGrab due to strain-specific glycan remodeling mechanisms (Sacher et al., 2020). Still, the example of FlaGrab points out the promises of phage proteins as potential therapeutic agents against pathogenic bacteria specifically targeting glycans or other (glycosylated) bacterial structures.

#### Flagellar Glycosylation of *C. difficile*

**Figure 3:**
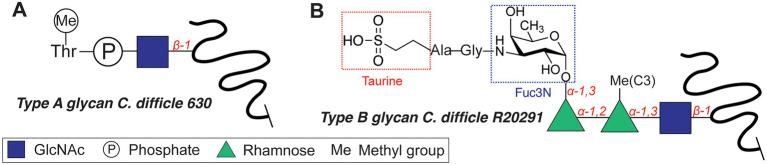
Flagellar glycan structures of *Clostridium difficile*. **(A)** Type A glycan. **(B)** Type B glycan.

The motility of *C. difficile*, an opportunistic Gram-positive pathogen, is dependent on *O*-glycosylation of its flagellum protein FliC. *C. difficile* strains display two different glycan structures (Twine et al., 2009; Faulds-Pain et al., 2014), with the core *N*-acetyl-β-glucosamine (β-GlcNAc) as the only conserved residue (Figure 3). NMR studies revealed that the type A *O*-glycan of the *C. difficile* 630 (Figure 3A) is composed of the core β-GlcNAc residue modified with an *N*-methylated Thr *via* a phosphate at the C3 position (Faulds-Pain et al., 2014). The more complex type B flagellar glycosylation (in strains BI-I, NAP-I, and ribotype 027) is composed of a Ser/Thr-linked β-GlcNAc, elongated with two rhamnose residues (*O*-methylated at the C3 position, Figure 3B; Bouché et al., 2016). An alternative structure featured an additional 3-amino-3-deoxy-d-fucose (Fuc3N in Figure 3B) modified with a sulfopeptide (Gly-Ala-taurine) at the C3-amino group (Bouché et al., 2016). The glycosyltransferases involved in the synthesis of the type B glycan include GT1 (core GlcNAc transfer onto Ser/Thr), bifunctional GT2 (Rha transfer and Rha methylation), and GT3 (partially involved in the synthesis of the sulfopeptide; Valiente et al., 2016). *C. difficile* knockout strains of GT1 and GT2 both resulted in decreased motility of the bacterium, whereas a GT1 deletion mutant showed only reduced adherence. These enzymes are interesting targets for antivirulence strategies, as the type B glycan is increasingly associated with the emerging hypervirulent and more aggressive strains of *C. difficile* (*e.g.* RT027, RT023).

### Immune Evasion

#### Capsular Polysaccharides

Capsular polysaccharides (CPS; Figure 1B) of Gram-negative and Gram-positive bacteria are constituents of the bacterial glycocalyx, providing protection to immune system recognition. Interestingly, the CPS of bacterial pathogens are often found to contain carbohydrate epitopes that mimic those of human cells which help to evade the immune system and promote infection (Cress et al., 2014). Consequently, encapsulated bacterial pathogens tend to be more virulent as they are less susceptible to immune system recognition and penetration of the antibiotics. Rendering bacterial pathogens non-encapsulated is an attractive prospect, as it would make the bacteria vulnerable to the innate immune response or resensitize the resistant strains to antibacterial treatments.

The CPS of pathogenic *E. coli* (so-called K capsules or K antigens) display highly diverse glycan structures, and ~80 different CPS are reported and classified into four groups depending on their assembly and export machinery (Whitfield, 2006). An interactive overview of the *E. coli* K antigens, their structures, and 3D modeling can be found in the *E. coli* K antigen 3D structure Database (EK3D; Kunduru et al., 2016).^1^

**Figure 4:**
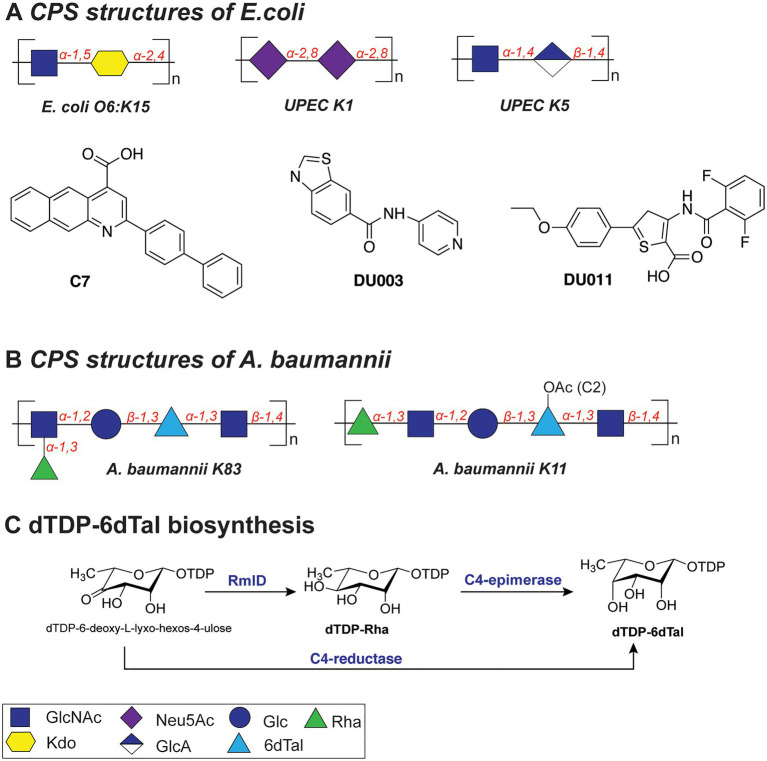
**(A)** Structures of the capsular polysaccharides of *Escherichia coli* and inhibitors described in this section. **(B)** Structures of the capsular polysaccharides of *Acinetobacter baumannii*. **(C)** Biosynthesis of dTDP-6dTal.

For example, the K15 antigen (Figure 4A) of enterotoxigenic *E. coli* O6:K15 is a polymer containing α-GlcNAc(1➔5)-α-Kdo(2➔4) disaccharide repeating units (Azurmendi et al., 2020), the K1 capsule (Figure 4A) of uropathogenic *E. coli* (UPEC) is composed of α-2,8-Neu5Ac repeats, and the K5 capsule is a polymer of α*-*GlcNAc(1➔4)*-*β-GlcA(1➔4) repeats (Figure 4A). The highly acidic capsule polysaccharides enhance bacterial survival by sequestering the antimicrobial peptides produced by the host immune system. A capsule-specific phage screen was used to identify inhibitors of CPS synthesis of UPEC K1 and K5 capsules (Goller and Seed, 2010). The most potent compound was 2-(4-phenylphenyl)benzo[g]quionoline-4-carboxylic acid (also called “C7,” Figure 4A), which showed an IC_50_ value of 12.5–25μM with UPEC K1 UTI89. C7 was evaluated in a variety of biochemical studies and was shown to specifically disrupt the oligomerization of the *E. coli* K1 antigen leading to its absence on the outside of the cell. Importantly, upon treatment with C7, the *E. coli* cells were more susceptible to human serum and the compound proved to be active also on clinical *E. coli* isolates. In a follow-up study, compounds DU003 and DU011 (Figure 4A) were identified from a structurally diverse set of small-molecule inhibitors, because they improved pharmacological properties (IC_50_, solubility, toxicity, permeability and plasma stability; Goller et al., 2014). Compound DU011 was later shown to attenuate CPS production in *E. coli via* interaction with the multi-drug efflux pump transcriptional regulator MprA (Arshad et al., 2016). Notably, this mode of inhibition was found to be antivirulent in nature, as it did not lead to the development of antibiotic resistance (Arshad et al., 2016).

A different strategy to de-encapsulate *E. coli* is based on phage-derived polysaccharide depolymerases (Lin et al., 2017). Several depolymerases were tested for their *in vitro* and *in vivo* (mouse models) activity with depolymerase enzyme K5 displaying the highest efficacy and consequent survival of mice. Importantly, the enzymes tested in this study (K1E, K1F, K1H, K5, and K30) were not toxic when injected in animals (based on survival, behavior and body weight monitoring for 5days). Furthermore, when *E. coli* was tested in a serum sensitivity assay, in the presence of depolymerases, the viability of the cells was reduced significantly, with K5 depolymerase displaying the most pronounced effect. The high specificity of depolymerases toward certain CPS structures represents a potential novel narrow-spectrum treatment.

The capsules of *Acinetobacter baumannii* are the main virulence factor of these bacterial species (Harding et al., 2017). Their CPS structures feature an impressive diversity of monosaccharides in repeating units and linkages, all of which complicate the development of treatments, especially vaccines (Singh et al., 2019). For example, a study of the association of different *A. baumannii* capsule types with carbapenem resistance revealed four main serotypes that contribute to resistance (KL2, KL10, KL22, and KL52), indicative of the importance of capsule structure in infection (Hsieh et al., 2020).

The repeating K units of *A. baumannii* CPS typically consist of 2–6 monosaccharide units and feature glucose, galactose, glucuronic acid, and nonulosonic acid, among others, also with acetyl or acyl modifications (K83 and K11; Figure 4B; Singh et al., 2019). Interestingly, several clinical isolates of *A. baumannii* (strains KL106, KL112, 48-1789, MAR24) were found to contain the bacterial monosaccharides 6-deoxy-l-talose and l-rhamnose (Kenyon et al., 2017; Kasimova et al., 2021). Importantly, dTDP-6-deoxy-l-talose is produced either from dTDP-l-Rha by the action of C4-epimerase or from dTDP-6-deoxy-l-lyxo-hexos-4-ulose by C4-reductase (Figure 4C; Kenyon et al., 2017). Consequently, the disruption of the dTDP-l-Rha biosynthesis pathway (*rmlABCD* cluster) can potentially abolish the synthesis of both rare monosaccharides in *A. baumannii*.

#### Lipooligosaccharides of *Neisseria gonorrhoeae* and *C. jejuni*

Lipooligosaccharides (LOS) are a major family of glycolipids presented on the outer membrane of the Gram-negative bacteria, and they play central roles in the virulence of many pathogens such as *Neisseria gonorrhoeae* and *C. jejuni.* Among other functions, LOS may aid pathogens in evading the host immune system or conferring immune resistance (Preston et al., 1996; Harvey et al., 2000; Song et al., 2000).

**Figure 5:**
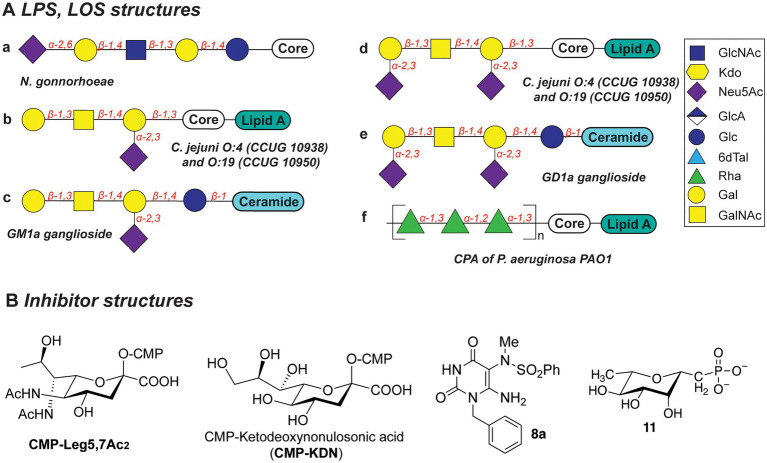
**(A)** Structures of LOS described in this section. **(B)** Structures of inhibitors discussed in this section.

Several *N. gonorrhoeae* strains express the tetrasaccharide lacto-*N*-neotetraose (LNnT) at their LOS termini which mimics the terminal glycan structure of the human glycosphingolipid precursor paragloboside (Mandrell et al., 1988; Tsai and Civin, 1991). The LNnT termini of LOS can be sialylated with *N*-acetylneuraminic acid (Neu5Ac) by the *N. gonorrhoeae* sialyltransferase LsT (Figure 5Aa; Mandrell et al., 1990, 1993; Gulati et al., 2005; Packiam et al., 2006). The sialylated LNnT motif confers resistance to the bactericidal effect of the complement system, a trait also denoted as “serum resistance,” and enables immune evasion (Mandrell et al., 1990; Wetzler et al., 1992; Ram et al., 1998, 2017; Gulati et al., 2005; Ricklin et al., 2010). Due to its key role in the establishment and maintenance of an infection by immune evasion, LsT of *N. gonorrhoeae* is therefore an attractive target for antivirulence intervention. In recent studies (Gulati et al., 2015), several CMP-nonulosonate analogues were identified which partly inhibited serum resistance and relieved the burden of gonococcal infection in mice models. Of the identified CMP-nonulosonate analogues, CMP-Leg5,7Ac_2_ and CMP-ketodeoxynonulosonate (CMP-Kdn; Figure 5B) were found to be most promising as future therapeutics (Gulati et al., 2020). Both compounds were stable in an acidic environment mimicking the human vaginal site of infection. Furthermore, they effectively treated infection with multi-drug-resistant gonococci in mice models presenting a humanized sialome or expressing a humanized complement system. Thus, CMP-Leg5,7Ac_2_ and CMP-Kdn are promising candidates for future therapeutics against multi-drug resistant *N. gonorrhoeae* strains. Interestingly, their mode of action follows the mechanism of metabolic oligosaccharide engineering (MOE), as will be discussed in section “Promising Strategies to Abolish Bacterial Glycosylation Systems”. Interestingly, a recent study identified an alternative terminal epitope of LNnT, featuring a Kdo residue that was transferred by the sialyltransLsT (Jen et al., 2021). This specific LOS structure was identified in the clinical isolates of *N. gonorrhoeae* and shown to be recognized by anti-Kdo monoclonal antibody 6E4 with potential for the future vaccine development.

Biosynthesis of the LOS core of *Campylobacter jejuni* is performed by a series of carbohydrate biosynthesis and glycosyltransferase enzymes. Whereas the inner LOS core of *C. jejuni* (which contains two heptose and two glucose moieties) is conserved (Klena et al., 1998; Gilbert et al., 2002; Kanipes et al., 2004, 2006), the outer core LOS is highly variable among *C. jejuni* strains. For example, the outer LOS core of *C. jejuni* strains CCUG 10938 and 10,950 contains the monosaccharides Gal, GalNAc, and Neu5Ac, which together resemble the terminal saccharides of host gangliosides GM1 or GD1a (Figures 5Ab-e; Yuki et al., 2004; Goodfellow et al., 2005; Godschalk et al., 2007; Janssen et al., 2008; Jasti et al., 2016). Bacterial strains that express the enzymes to produce these host-mimicking epitopes have been linked to the development of the autoimmune Guillain-Barré syndrome (GBS) wherein autoantibodies induce damage to nerve gangliosides (Nachamkin et al., 2002; Godschalk et al., 2004; Mortensen et al., 2009; Poole et al., 2018). Counteracting the molecular mimicry of *C. jejuni* is therefore of interest for reducing immune evasion and severity of GBS following a *C. jejuni* infection. Inhibition of the glycosyltransferases that are required to build the core LOS glycans of *C. jejuni* would be an effective way to preclude the immune evasion caused by molecular mimicry of *C. jejuni*.

#### Lipopolysaccharides of *P. aeruginosa*

The Gram-negative bacterial pathogen *P. aeruginosa* produces two main types of the lipopolysaccharides: common polysaccharide antigen (CPA) with d-Rha repeats as an outer core (Figure 5Af) and O-specific antigen (OSA) with varied structures across 20 serotypes (heteropolymer of 2–4 monosaccharides: GalNAc, GlcNAc, ManNAc, QuiNAc, GulNAc, FucNAc, l-Rha, Xyl, among others; Lam et al., 2011). Both types of LPS are important for the pathogenesis and survival of *P. aeruginosa*, as they confer serum resistance, prevent phagocytosis, and promote swimming and swarming motility and biofilm formation (Huszczynski et al., 2020).

Due to the importance of rhamnose in the infection strategies of *P. aeruginosa*, the dTDP-l-Rha donor synthesis pathway is an attractive antibiotic target. Several studies explored substrate mimics as inhibitors of the TDP-l-Rha biosynthesis. For example, thymidine-based allosteric inhibitors have been developed for RmlA, the first enzyme in the TDP-Rha synthesis that performs condensation of the glucose-1-phosphate (G1P) with thymidine triphosphate (dTTP; Alphey et al., 2013). The library screen and subsequent optimization of the lead compounds yielded a potent inhibitor (called “8a,” Figure 5B) with IC_50_ of 0.073μM, as determined *in vitro*. Importantly, *via* X-ray structure determination and SPR analyses, the authors deduced that 8a acts as a competitive allosteric inhibitor of glucose-1-phosphate binding. The allosteric site of the RmlA plays a role of inducing a negative feedback loop of the TDP-Rha synthesis upon binding the TDP-Rha. It is hypothesized that binding of 8a locks the tetrameric enzyme in a fixed conformation, which prevents G1P binding in the active site.

In a different study, a panel of l-rhamnose 1C-phosphonate and (fluorinated) ketosephosphonate compounds was prepared and evaluated as inhibitors of the TDP-l-Rha biosynthesis enzymes from *P. aeruginosa* and *Streptococcus pneumoniae* (Loranger et al., 2013). l-rhamnose 1C-phosphonate (called “11,” Figure 5B) was determined to be the best with an IC_50_ of 5.7mM. Compound 11 is expected to behave as a competitive inhibitor of the G1P binding in the active site of Cps2L of *S. pneumoniae* (RmlA in *P. aeruginosa*), and addition of a thymidine moiety may improve the potency of the inhibitor.

### Biofilms

Bacterial biofilms are complex entities comprised of aggregated bacterial cells enclosed in a secreted matrix that contains polysaccharides, proteins, lipids, and extracellular DNA and typically attached to the (a)biotic surfaces. Biofilms are characterized by increased resistance to antimicrobials and subsequent enhanced survival of bacteria in the biofilm and feature a distinct metabolic and genetic makeup (Flemming et al., 2016). Microbial biofilms are of great concern in the context of hospital infections (especially *via* medical devices), and multiple methods have been developed to study and mimic biofilm formation (Azeredo et al., 2017). Antibiotic resistance conferred by biofilms results in recurring or chronic infections which are responsible for a great personal and healthcare burden (Sharma et al., 2019). Therefore, methods to prevent or disrupt the biofilms are of great importance. A plethora of small-molecule therapeutics, enzymes and physical methods for biofilm prevention, inhibition, and dissemination have been developed (Verderosa et al., 2019; Ghosh et al., 2020; Pinto et al., 2020).

**Figure 6:**
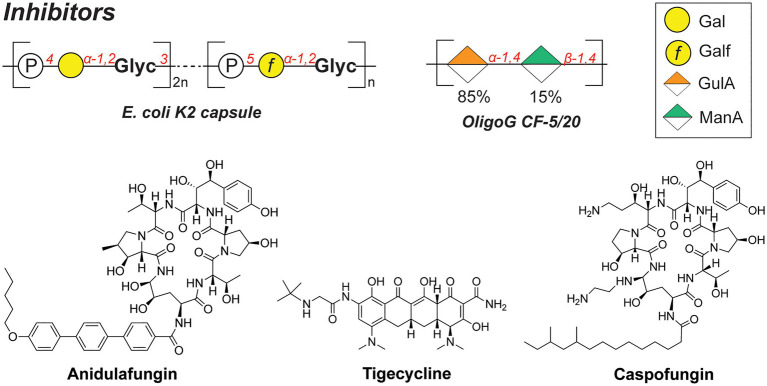
Structures of biofilm inhibitors described in this section.

Whereas bacterial capsules generally confer enhanced survival for encapsulated pathogens (as described above), they also display inhibitory properties toward competing microbial species. For instance, a soluble polysaccharide (K2 capsule, Figure 6) secreted by UPEC was found to have anti-adhesive properties that preclude biofilm formation of both Gram-negative (*E. coli*, *K. pneumoniae*, and *P. aeruginosa*) and Gram-positive species (*Staphylococcus aureus*, *Staphylococcus epidermidis*, and *Enterococcus faecalis*; Valle et al., 2006). The released CPS of UPEC tested in the study were shown to reduce the initial cell surface contacts and interfere with the cell–cell aggregation, both processes necessary for the biofilm formation. Importantly, a full-length polysaccharide was required to confer the inhibitory properties, as hydrolyzed polymer did not exert the same effect.

Similarly, the alginate oligomer OligoG [α*-*GulA(1➔4)*-*β-ManA(1➔4), Figure 6], currently in stage 2b clinical trials for cystic fibrosis treatment, was found to dissolve the biofilms of mucoid *P. aeruginosa* (Hengzhuang et al., 2016). The low-molecular weight OligoG *CF*-5/20 (purified from seaweed *Laminaria hyperborea,* 85% GulA, and 15% ManA content, ~3,200Da, DP=16) showed synergistic effects when combined with the antibiotic colistin in a murine lung infection model. A follow-up study revealed that OligoG interacts with components of the *P. aeruginosa* EPS, penetrating into the biofilm and disrupting the Ca^2+^-eDNA complexes involved in the biofilm maturation process (Powell et al., 2018).

Interestingly, biofilms frequently feature multi-species communities which complicate the development of effective antibacterial therapies. Consequently, combination therapies and drug adjuvants are a promising strategy to target and eradicate several (bacterial) pathogens simultaneously. For instance, the joint use of antifungal and antibacterial compounds was recently reported to effectively disperse a *Candida albicans*-*S. aureus* biofilm (Rogiers et al., 2018). These species are postulated to have a mutualistic relationship, specifically in the context of intra-abdominal infections (IAIs). In addition, a combination therapy of anidulafungin (against *C. albicans*; Figure 6) and tigecycline (against *S. aureus*; Figure 6) on a dual-species biofilm in the IAI murine model showed a synergistic effect, eradicating *S. aureus* more effectively compared to the treatment with tigecycline alone. Increased administration of anidulafungin resulted in the reduced presence of poly-β-1,6-*N*-acetylglucosamine (PNAG) which is a major polysaccharide constituent of the *S. aureus* biofilm EPS. It was hypothesized that the mode of action of anidulafungin parallels the action of caspofungin, which was previously reported to disrupt the function of the PNAG-synthesizing *N*-acetylglucosamine transferase IcA (Siala et al., 2016). When used as an adjuvant with fluoroquinolones, which are typically used to treat *S. aureus* infections, anidulafungin showed a marked synergistic effect, resulting in enhanced penetration of fluoroquinolones into the biofilm, possibly due to the decreased PNAG presence.

### Exotoxins

**Figure 7:**
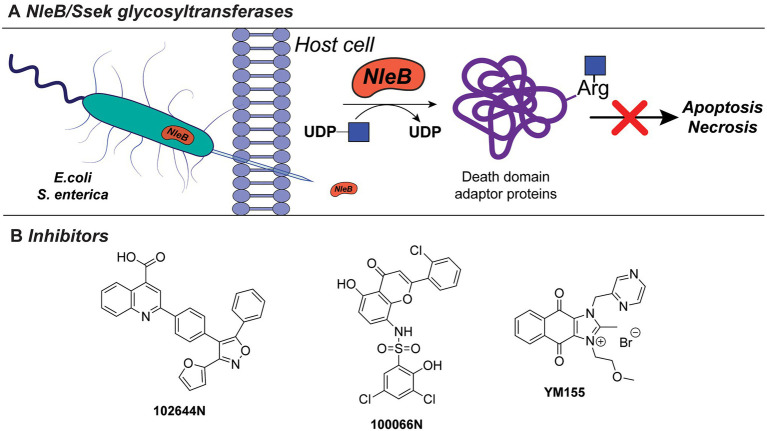
**(A)** NleB/Ssek exotoxin system. **(B)** Developed inhibitors against NleB/Ssek discussed in this section.

Bacterial pathogens actively modulate host immune and tissue cells processes to evade recognition and promote survival and spread in the host (Sastalla et al., 2016). This is achieved, for instance, *via* the secretion of bacterial exotoxins with glycosyltransferase activity which alters or disrupt specific host processes (Sastalla et al., 2016). For example, NleB1 of enterohaemorrhagic *E. coli* (EHEC), enteropathogenic *E. coli* (EPEC), and *Citrobacter rodentum*, and SseK of *Salmonella enterica* are conserved glycosyltransferase effectors, that are injected into the host cells by a type III secretion system (Figure 7A). These exotoxins transfer β-GlcNAc to arginine residues on host cell proteins, such as serine/threonine-protein kinase 1 (RIPK1), tumor necrosis factor receptor (TNFR) type 1-associated DEATH domain protein (TRADD), the Fas-associated protein with death domain (FADD), and the mammalian glycolysis enzyme glyceraldehyde 3-phosphate dehydrogenase (GAPDH). Glycosylation of these target proteins results in the inhibition of innate host immune responses facilitating spread and host cell infection (Gao et al., 2013; Li et al., 2013; Pearson et al., 2013; Esposito et al., 2018; Park et al., 2018).

Inhibitors directed against NleB1, as well as the *S. enterica* analogues Ssek1 and Ssek2, have been identified and showed promising results as new antivirulence agents. In a recent study, a small-scale high-throughput screen for inhibitors of NleB1 of EPEC and EHEC was performed using a library of 5,160 small-molecule compounds (El Qaidi et al., 2018). Using this setup, two compounds, 100066N and 102644N (Figure 7B), were found to inhibit Nleb1 and SseK1/SseK2 activity *in vitro*, as well as NleB1 activity on mammalian HEK293 cells. The compounds inhibited replication of *S. enterica* strain ATCC 14028 in mouse macrophage-like cell infection assays, while they were not cross-reactive toward mammalian *O*-linked *N*-acetylglucosaminyltransferases (OGT) and did not inhibit growth of *S. enterica* bacterial cultures indicating that they are not bactericidal (El Qaidi et al., 2018). Since compounds 100066N and 102644N have relatively low solubilities and are not commercially available, a library screen of 42,498 compounds, containing more diverse chemical scaffolds with favorable characteristics for future chemical optimization, was performed. In this new screen, the commercially available compound sepantronium bromide (YM155, Figure 7B) was found to robustly inhibit NleB/SseK glycosyltransferases. YM155 was previously described as a small-molecule inhibitor of survivin, which belongs to the inhibitor of apoptosis (IAP) protein family (Ambrosini et al., 1997; Nakahara et al., 2007). While the inhibition of NleB/SseK is concentration-dependent, YM155 did not cross-react with the human OGT enzyme, supporting its specificity to NleB/SseK glycosyltransferases. In addition, YM155 did not exhibit toxicity in RAW264.7 cells (Zhu et al., 2021). However, the effect of YM155-mediated inhibition on survivin has not been characterized in the study. The growth of *C. rodentium,* EHEC, or *S. enterica* cultures was not significantly altered at maximum concentrations of YM155 (125μM). Furthermore, treatment of macrophage RAW264.7 cells with YM155 reduced the amount of infected, intracellular bacteria as quantified by *Salmonella* infection assays. Compared to 100066N and 102644N, YM155 is less potent, but showed higher solubility and is easier to chemically modify for future structural improvements. Together with its commercial availability, YM155 poses an interesting candidate for further characterization and chemical modification and development into a future antivirulence drug.

#### TcdA/B Toxins of *C. difficile*

**Figure 8:**
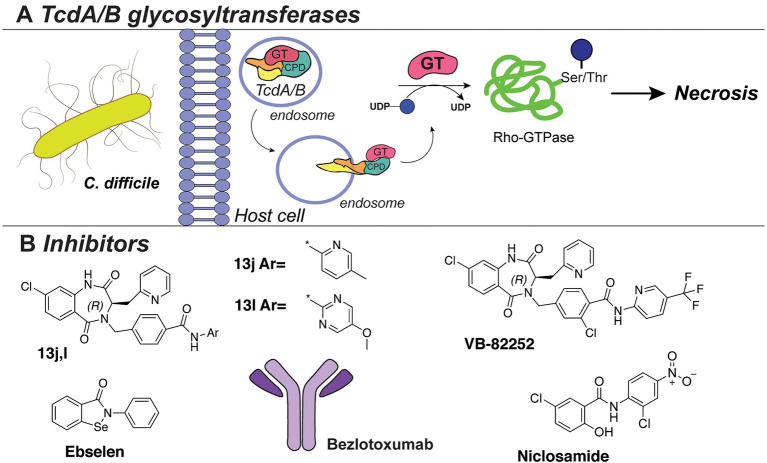
**(A)** The TcdA/TcdB bacterial exotoxin system. **(B)** Inhibitors against TcdB discussed in this section.

One of the best-studied examples of bacterial cytotoxins is TcdA and TcdB of the opportunistic pathogen *C. difficile*, with TcdB expressed predominantly in hypervirulent strains (Figure 8A). These clostridial toxins are the main determinants of bacterial pathogenesis, as they form pores in the host cells and modulate cell death, thereby spreading the infection. TcdA/TcdB toxins are composed of four domains, namely, transporter domain, receptor-binding domain, cysteine protease domain (CPD), and an N-terminal glycosyltransferase domain (GT; Di Bella et al., 2016; Aktories et al., 2017). Upon acidic endocytosis into the host cell, the toxins are translocated through the membrane where the CDP domain catalyzes cleavage and release of the N-terminal GT domain (Figure 8A; Di Bella et al., 2016). Subsequently, the GT domain transfers d-glucose onto threonine residues of host cell Rho-guanosine triphosphatases (Rho-GTPase; Just et al., 1995a,b; Kuehne et al., 2010). This leads to necrosis characterized by cell rounding, membrane blobbing, and finally, cell death (Sehr et al., 1998; Genth et al., 1999; Voth and Ballard, 2005).

Several HTS studies were performed to identify small-molecule inhibitors of *C. difficile* toxins. A screen of six million compounds, followed by extensive optimization of the lead compounds *via* chemical synthesis and SAR analysis, yielded compounds “13j” and “13l” (Figure 8B). These compounds share a benzodiazepinedione core and displayed potent inhibitory activity against TcdB (low nM IC_50_ *in vitro*, low μM in a cell assay; Letourneau et al., 2018). Interestingly, the compounds were not bactericidal to *C. difficile* or gut bacteria. However, all potent compounds demonstrated low mouse plasma stability and rapid clearance. The same research group also reported the biological evaluation of compound VB-82252 (Figure 8B), which exhibited low plasma stability, but high oral bioavailability (Stroke et al., 2018). Compound VB-82252 was found to be a potent inhibitor (IC_50_ of 32nM) of UDP-Glc hydrolysis by TcdB (used as a measure of TcdB activity), as determined in an *in vitro* assay (Stroke et al., 2018). The compound was effective in preventing CHO cells rounding in an assay with several strains of *C. difficile*. The therapeutic efficiency of VB-82252 was further evaluated in a mouse and hamster *C. difficile* disease model, where it was effective in sustaining body weight and prolonging the survival of the animals.

In addition, the cell rounding assay was also employed to quantify the effects of various approved therapeutics on TcdB toxins inhibition (Tam et al., 2018). From this library screen assay, the drug niclosamide (Figure 8B), originally developed to treat GI parasites, was most potent (EC_50_~0.5 μM) in protecting the human cells from rounding. Interestingly, niclosamide does not inhibit the TcdB toxin directly, but instead increases the pH of the host endosomes which disrupts the toxin uptake into the cells. Treatment with niclosamide was effective in a murine model of infection while it exhibited no bactericidal effect on *C. difficile* or beneficial gut bacteria (determined with an MIC assay and diversity monitoring, respectively).

In an alternative approach to TcdA/B inhibition, small-molecule inhibitors of the cysteine protease domain (CPD) of the *C. difficile* toxin were identified (Bender et al., 2015). By utilizing fluorescence-polarization HTS of compound libraries with clinically safe drugs (*e.g.*, LOPAC library), multiple inhibitors were identified, with the selenium-containing drug ebselen (Figure 8B) exhibiting the highest potency (IC_50_ 6.9nM). Notably, the drug could preclude the GT domain release and cell rounding. The effects of the drug were confirmed to be due to the prevention of Rho-GTPases glucosylation, and it was shown to be effective in a murine model of *C. difficile* infection. Importantly, ebselen is a developed drug in late clinical trials for the treatment of tinnitus, hearing loss, and bipolar disorder and has been proven safe for use in humans.

Besides conventional antibiotics to treat a *C. difficile* infection, the TcdB-neutralizing antibody bezlotoxumab is an FDA-approved therapy against recurring *C. difficile* (Zinplava, Merck; Navalkele and Chopra, 2018). Bezlotoxumab binds the N-terminal part of the receptor-binding domain of the TcdB toxin, preventing toxin binding and entry into host cells (Orth et al., 2014). It was also effective against hypervirulent *C. difficile* strains (NAP1, BI, 027). Currently, bezlotoxumab is only used in combination with antibiotic treatments and is not a stand-alone therapy against *C. difficile*.

## Part 2: Future Perspectives

### Bacterial Protein Glycosylation Systems as Promising Targets

#### Adhesins and Autotransporters

Adhesion is one of the first step in the bacterial colonization of the host. It is mediated by various adhesion factors presented on the surface of the bacterium that recognize and bind to the host cell receptors (Chagnot et al., 2013; Poole et al., 2018). Adhesin proteins in particular are often (hyper)glycosylated, and the presence of glycans often plays a vital role for their stability and proper function (Lu et al., 2015). Therefore, glycosylation of the adhesion factors is an attractive target for the development of novel anti-adhesive therapies.

#### *O*-Heptosylation of the Self-Associating Autotransporters

Diffusely adhering *E. coli* (DAEC), enterotoxigenic *E. coli* (ETEC), and the murine pathogen *Citrobacter rodentium* share a common adhesion mechanism to host cells. These bacteria rely on a type Va secretion system, which is also called a self-associating autotransporter (SAAT) system (Lu et al., 2014). Autotransporter proteins consist of a C-terminal β-barrel domain that forms a transport channel in the outer membrane and a passenger domain which is translocated through this channel and fulfils the effector adhesion function (Leyton et al., 2012). In DAEC, ETEC, and *C. rodentium,* the passenger domains of autotransporters AIDA-I, TibA, and CARC, respectively, are *O*-hyperglycosylated with bacteria-specific d-glycero-d-manno-heptose (ddManHep Figure 1) by a cognate GT belonging to the *b*acterial *a*utotransporter *h*eptosyl*t*ransferase (BAHT) family (Lu et al., 2014, 2015). Hyperglycosylation is important for the successful adherence of AIDA-I to HeLa cells (Benz and Schmidt, 2001) and was later found to enhance protein stability (Charbonneau et al., 2007). Similarly, TibA is the SAAT of enterotoxigenic *E. coli* and depends on hyperheptosylation for stability as it was found to mediate its (re)folding and subsequently influence adherence function (Côté et al., 2013). Interestingly, heptose residues also constitute the LPS core of Gram-negative bacteria, and the synthesis pathway of ADP-l-glycero-β-d-manno-heptose (Kneidinger et al., 2002) is considered a promising target for inhibitors. Several studies have already identified inhibitors with IC_50_ values in the milli/micromolar range (De Leon et al., 2006; Kim et al., 2021a,b). It would be interesting to investigate whether these inhibitors indeed abolish SAAT hyperheptosylation and subsequent adherence of the bacterial cells.

#### *N*-Glycosylation of HMW Adhesins and Trimeric Autotransporters

Non-typeable *Haemophilus influenzae* (NTHi) utilizes a type Vb secretion system (also called two-partner secretion (TPS) pathway) to transport and present high molecular weight (HMW) adhesin proteins on the surface as a first step in host colonization (St Geme et al., 1993; Grass and St Geme, 2000). Stability and efficient surface tethering of HMW adhesins is dependent on *N*-hyperglycosylation on asparagine with simple mono- and disaccharides of glucose (Glc; Grass et al., 2003). A total of 31 glycosylation sites have been identified at asparagine residues in the Asn-X-Ser/Thr consensus sequence of HMW1A (Grass et al., 2003, 2010; Gross et al., 2008), modified by the action of the associated glycosyltransferase HMW1C (Grass et al., 2010). Interestingly, the glycosylation of HMW1A by HMW1C follows an unconventional OTase-independent *N-*glycosylation pathway, wherein cytoplasmic HMW1C transfers single nucleotide-activated carbohydrates to the acceptor protein HMW1A. Upon deletion of the genes encoding for HMW1C and UDP-Glc biosynthesis, *hmw1c* or *galU,* respectively, HMW1A surface presentation as well as adhesion to epithelial cells was abolished *in vitro* (Grass et al., 2010). Interestingly, we recently revealed that hyperglycosylation is established through a semiprocessive mechanism *in vitro* (Yakovlieva et al., 2021). Homologues of the HMW1C glycosyltransferase have been identified in *Kingella kingae* (HMW1C_Kk_) and *Aggregatibacter aphrophilus* (HMW1C_Aa_) where they perform the glycosylation of cognate trimeric autotransporters Knh and EmaA, respectively (Rempe et al., 2015). Analogously to the *H. influenzae* HMW1A, abolishing glycosylation of Knh and EmaA was shown to inhibit the bacterial aggregation and adherence to the host cells.

#### O-Glycosylation of Serine-Rich Repeat Proteins of Gram-Positive Bacteria

Multiple members of the serine-rich repeat proteins (SRRPs) of clinically relevant Gram-positive bacteria are found to be (hyper) *O*-glycosylated with carbohydrates that influence stability and adhesive function. Examples include fimbriae-associated protein Fap1 of *Streptococcus parasanguinis*, GspB of *Streptococcus gordonii*, SraP of *S. aureus*, PsrP of *S. pneumoniae,* and others, which are reviewed elsewhere (Zhou and Wu, 2009; Lizcano et al., 2012). Glycosyltransferases termed Gtf1-Gtf2 (GtfA-GtfB) are responsible for the core GlcNAc modification on the Ser/Thr residues of SRRPs. These enzyme pairs operate in tandem with Gtf1 performing the glycosylation reaction and Gtf2 acting as a chaperone and substrate-binding domain (Wu and Wu, 2011; Chen et al., 2016; Zhao et al., 2018). After attachment of the initial GlcNAc, the character of the glycan modifications varies between different SRRPs and features GlcNAc/Glc (Srr1, GspB; Bensing et al., 2004; Chaze et al., 2014), Glc/GlcNAc/Rha (Fap1; Zhu et al., 2016), and GlcNAc (SraP; Li et al., 2014). The presence of the multiple glycans on the SRRPs is crucial for their function in conferring adhesion and biofilm formation of the Gram-positive pathogens and therefore constitutes an interesting antibacterial target.

#### Glycosylation of Pili

*Neisseria gonorrhoeae* produces type IV pili (TFP; Patel et al., 1991) that are required for effective adhesion to epithelial cells in an initial stage of the infection (Swanson, 1973; McGee and Stephens, 1984; Virji and Heckels, 1984; Craig et al., 2004). The glycans of TFP interact with complement receptor 3 (CR3), an innate pattern recognition receptor expressed on human cervical cells. The PilE subunits that make up the TFP feature glycans containing either a *N,N′*-diactetylbacillosamine (diNAcBac) or a galactose-modified diNAcBac (Gal(α1-3)diNAcBac) linked to serine residues (Jennings et al., 1998, 2011; Power et al., 2003; Hegge et al., 2004; Hartley et al., 2011). Only *N. gonorrhoeae* cells carrying the disaccharide Gal(α1-3)diNAcBac on their PilE proteins survive infection of the primary human cervical (Pex) cells, while TFP decorated with a single diNAcBac die within the cervical cells, even though they were found to be hyperinvasive. Glycosylation of PilE follows an OTase-dependent *O*-glycosylation pathway by multiple pilin glycosylation genes (pgl), which encode enzymes for the synthesis and attachment of diNAcBac to an intermediate lipid carrier, as well as GTs that attach galactose or glucose to diBacNAc (Hartley et al., 2011). Considering the importance of PilE glycosylation in the colonization capacity of *N. gonorrhoeae*, inhibitors of the pgl enzymes are attractive antibacterial agents. However, no inhibitors of *N. gonorrhoeae* pgl enzymes have been reported to date. Nonetheless, there are alternative approaches for targeting the interaction between the glycosylated TFP and CR3. Recently, two clinically approved drugs have been identified that inhibit the interaction of glycosylated PilE of *N. gonorrhoeae* with the l-domain of CR3. The drugs carbamazepine and methyldopa act as competitive inhibitors of CR3 binding and thereby efficiently blocked *N. gonorrhoeae* infection in Pex cells. Importantly, both drugs were also effective against multi-drug resistant gonococci and did not lead to development of resistance.

#### Efflux Pump Glycosylation

Efflux pumps are membrane proteins involved in the transport of various molecules (Alcalde-Rico et al., 2016). In bacterial pathogens, efflux pumps are often responsible for ejecting antibiotics from bacterial cells. They are especially prominent in the multi-drug resistance species and are an attractive drug target, especially in combination therapies (Ferrer-Espada et al., 2019; Marshall et al., 2020; Rodrigues et al., 2020).

In *C. jejuni*, the CmeABC complex is the main multi-drug efflux pump that confers resistance to various antibiotics. Together, CmeA, CmeB, and CmeC form a superstructure that spans the inner membrane, periplasmic space and creates a pore in the outer membrane of the bacterial cell. It was previously reported to be *N*-glycosylated with complex *C. jejuni N*-glycans (Abouelhadid et al., 2020). In a recent study, the importance of *N-*glycosylation for CmeABC efflux pump function was revealed (Dubb et al., 2020). Abolishing glycosylation of CmeA, which spans the periplasmic space, led to the increased accumulation of ethidium bromide and significant increase in antibiotic susceptibility, both indicating the impaired functioning of the efflux pump machinery. Additionally, the loss of CmeA glycosylation resulted in the loss of colonization ability of the chicken ceca.

### Promising Strategies to Abolish Bacterial Glycosylation Systems

#### Inhibition by Metabolic Oligosaccharide Engineering

With the increasing knowledge of bacterial glycosylation systems, a variety of strategies to inhibit the enzymes involved has been developed. For instance, inhibitors of the enzymes involved in production of the carbohydrate-nucleotide donors, such as dTDP-Rha (*vide supra*), have been developed and described elsewhere (Alphey et al., 2013; Loranger et al., 2013; Van Der Beek et al., 2019). In addition, several classes of compounds have been designed as inhibitors for glycosyltransferases (Compain and Martin, 2001; Kajimoto and Node, 2009; Tedaldi and Wagner, 2014; Ema et al., 2018; Conforti and Marra, 2021). For this review, we decided to focus on the technique of MOE, as a promising strategy to interfere with bacterial glycosylation systems in a specific manner.

The technique of MOE, as originally developed by Reutter (Kayser et al., 1992) and Bertozzi (Mahal et al., 1997; Bertozzi and Saxon, 2000), relies on hijacking the cell’s own metabolism to introduce carbohydrate variants with altered properties. In this way, carbohydrate precursors carrying bioorthogonal handles can be introduced into the native glycans by permissive enzymes, allowing the subsequent attachment of reporter groups to detect carbohydrate incorporation and glycan production. While the technique was originally developed on eukaryotic cells, the interest in applying MOE to bacterial cells is steeply rising, and several bacterial glycans have now been targeted with unnatural carbohydrates (Tra and Dube, 2014; Clark et al., 2016).

**Figure 9:**
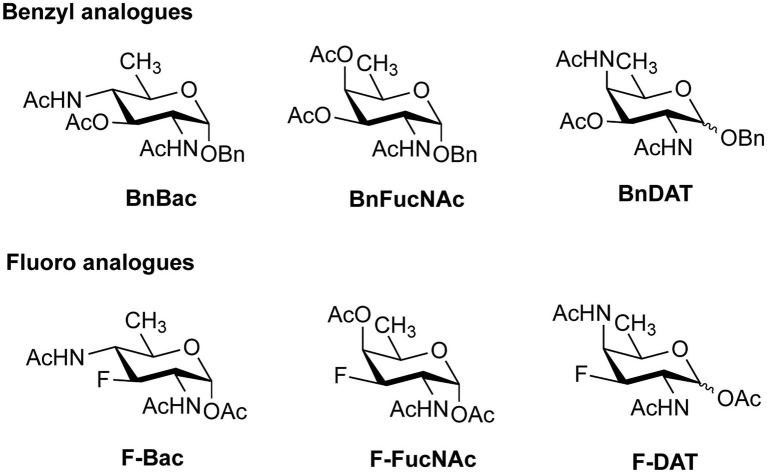
Benzyl and fluoro analogues designed for metabolic inhibition of glycosylation.

In addition to the promising application of labeling bacterial glycans for visualization, the MOE technique can also be used to introduce monosaccharide analogues that inhibit the proper assembly of bacterial glycans. To this end, both substrate decoys, which act as surrogate glycan acceptor sites (Dimitroff et al., 2003; Metastasis et al., 2009; Gloster and Vocadlo, 2012; Rillahan et al., 2012; Villalobos et al., 2015), and chain-terminating carbohydrate analogues, which lack a specific hydroxyl group for elongation (Li et al., 2016) have been developed for different bacterial strains. In a recent study, analogues of DiNAcBac, FucNAc, and DATDG were employed both as substrate decoys and inhibitors (Figure 9) to perturb glycan synthesis in *H. pylori* (Williams et al., 2020). The benzyl glycoside analogues BnBac, BnFucNAc, and BnDAT were synthesized as decoy substrates, and fluoro analogues F-Bac, F-FucNAc, and F-DAT were designed as chain-terminating inhibitors. Interestingly, treatment of *H. pylori* with BnBac, BnFucNAc, and F-DAT resulted in reduction of glycoprotein synthesis and defects in growth, biofilm formation, and motility. These functional defects could be largely reproduced in an isogenic *H. pylori* ∆GT mutant lacking a functional glycosylation system, proving that the MOE approach indeed has potential to be an antivirulence strategy. In addition, the analogues under study here also revealed bacteria-specific effects. In *C. jejuni*, none of the carbohydrate analogues impacted glycan biosynthesis or fitness, and only subtle changes were observed in the commensal *Bacteroides fragilis*. It will be interesting to test these carbohydrate analogues in animal models of infection and to understand their potential as narrow-spectrum antivirulence compounds.

## Discussion

Glycosylation is an intriguing feature of virtually all bacteria, and increasing amounts of evidence indicate that many bacteria rely on glycosylation for fitness and infection. As illustrated by the various examples in this review, especially pathogenic bacteria are often dependent on glycosylation of biomolecules related to virulence factors to successfully establish an infection. Whereas many virulence factors such as adhesins or flagella are glycosylated by internal GTs, exotoxins act as GTs themselves and actively modify molecular structures of the host organism to enable infection (Lu et al., 2015). Given the importance of virulence factors in the establishment of an infection, novel approaches that target glycosylation of virulence factors hold great promise as antibacterial strategy (Clatworthy et al., 2007). Several antivirulence agents have already been developed, and many are in (pre)clinical stages (Dickey et al., 2017); however, only few examples are specifically directed against bacterial glycans or glycosylation processes (*e.g.*, against TcdA/B; Dickey et al., 2017).

In this review, recent progress is highlighted in developing strategies to disturb and inhibit bacterial glycosylation enzymes and products, with a focus on antivirulence factors. While for some strategies the phenotypical effects are already validated on whole cells or infection models, others are still in the stage of proof of inhibition *ex vivo* (*e.g.*, on isolated enzymes). For instance, potent small-molecule inhibitors of diNAcBac biosynthesis in *C. jejuni* and inhibitors of GTs from *Neisseria* and *Haemophilus* have been developed, but they have not yet been tested or did not show a phenotypic effect in cell culture or *in vivo* models (De Schutter et al., 2017; Xu et al., 2017, 2018). A major challenge for small-molecule inhibitors of cytoplasmic targets, such as GTs or carbohydrate biosynthesis enzymes, is to pass the complex bacterial cell wall to gain cell entrance (Tiz et al., 2018). Indeed, most antivirulence drugs in advanced preclinical or clinical developmental stages act on surface-exposed or secreted virulence factors (Dickey et al., 2017). Various approaches have been developed to overcome the problem of cell wall permeability which include altering of physicochemical properties of the drugs, coupling drugs to siderophores, inhibiting efflux pumps, and using liposomes as drug carriers (Tiz et al., 2018). However, there is not a common consensus about universal rules facilitating drug penetration yet (Tiz et al., 2018). In addition to the challenge of target localization, the generation of inhibitors against carbohydrate-active enzymes is itself a daunting task. The high hydrophilicity of carbohydrate substrates and pyrophosphate moieties in nucleotide sugars warrant a creative approach to generate inhibitors that are also able to arrive at the target location (Merino et al., 2016). In case of glycosyltransferases, also the complex mechanism, in which multiple substrates are involved, complicates this process. The concept of bisubstrate-analogue inhibitors is a promising strategy (Kajimoto and Node, 2009), as is the development of bacteria-specific iminosugars (Conforti and Marra, 2021). Future developments in this area will facilitate the generation of small-molecule inhibitors of glycosylation enzymes with better cell wall penetration properties.

Metabolic inhibitors of bacterial glycan biosynthesis hold a promise to selectively target specific bacteria and their virulence factors (Williams et al., 2020). As the MOE technique relies on the peracetylated monosaccharide analogues, the compounds can successfully pass the bacterial cell membrane. Interestingly, a recent study reveals that these peracetylated carbohydrates may suffer from non-enzymatic *S*-glycosylation in living (eukaryotic) cells (Qin et al., 2018). Additional experiments to further investigate this side effect are needed to profile the occurrence of protein labeling and the impact on both bacterial and eukaryotic cells.

The sheer number of different monosaccharides that are identified in bacteria (Imperiali, 2019), and the certainty that this number will increase over time, makes the development of strategies to target the enzymes involved and their respective products a highly promising strategy to tackle the challenge of antibiotic resistance.

## Author Contributions

LY and JF contributed to the organization and structure of the review. LY, MW, and JF contributed to the writing and critical evaluation of the article. All authors contributed to the article and approved the submitted version.

## Funding

This work was financially supported by the Dutch Organization for Scientific Research (VENI 722.016.006) and the European Union through the Rosalind Franklin Fellowship COFUND project 60021 (both to MW).

## Conflict of Interest

The authors declare that the research was conducted in the absence of any commercial or financial relationships that could be construed as a potential conflict of interest.

## Publisher’s Note

All claims expressed in this article are solely those of the authors and do not necessarily represent those of their affiliated organizations, or those of the publisher, the editors and the reviewers. Any product that may be evaluated in this article, or claim that may be made by its manufacturer, is not guaranteed or endorsed by the publisher.

## Figures and Tables

**Table:** 

Term	Definitions
Ac	acetyl
ADP	adenosine diphosphate
Ala	alanine
Asn	asparagine
Bac	bacillosamine
BAHT	bacterial autotransporter heptosyltransferase
CMP	cytidine monophosphate
CPA	common polysaccharide antigen
CPD	cysteine protease domain
CPS	capsular polysaccharide
DAEC	diffusely adhering E. coli
DATDG	2,4-diacetamido-2,4,6-trideoxygalactose
diNAcBac	N, N'-diacetylbacillosamine
DNA	deoxyribonucleic acid
DP	degree of polymerization
dTDP	deoxythymidine diphosphate
dTDP-L-Rha	deoxythymidine diphosphate-L-rhamnose
EHEC	enterohaemorrhagic E. coli
ELISA	enzyme-linked immunosorbent assay
EPEC	enteropathogenic E. coli
EPS	extracellular polymeric substances
ETEC	enterotoxigenic E. coli
FDA	Food and Drug Administration
Fuc3N	3-amino-3-deoxy-D-fucose
FucNAc	N-acetylfucosamine
G1P	glucose-1-phosphate
GAPDH	glyceraldehyde 3-phosphate dehydrogenase
GBS	Guillain-Barré syndrome
GI	gastrointestinal
Glc	glucose
GlcA	glucuronic acid
GlcNAc	N-acetylglucosamine
Gly	glycine
GT	glycosyltransferase
GulNAc	N-acetylgulosamine
HMW	high molecular weight
HTS	high throughput
IAI	intra-abdominal infection
IAP	inhibitor of apoptosis
IC50	concentration to inhibit 50% enzyme activity
Kdo	3-deoxy-D-manno-octulosonic acid
Leg	legionaminic acid
LNnT	lacto-N-neotetraose
LOS	lipooligosaccharides
LPS	lipopolysaccharide
ManA	mannuronic acid
ManNAc	N-acetylmannosamine
MDR	multidrug resistance
MIC	minimal inhibitory concentration
MOE	metabolic oligosaccharide engineering
Neu5Ac	neuraminic acid
NTHi	nontypeable Haemophilus influenzae
OGT	O-GlcNAc transferase
OSA	O-specific antigen
OTase	oligosaccharyltransferase
PNAG	poly-β-1,6-N-acetylglucosamine
Pse	pseudaminic acid
QuiNAc	N-acetylquinosamine
Rha	L-rhamnose
Rho-GTPase	Rho-guanosine triphosphatases
RIPK1	serine-threonine protein kinase 1
SAAT	self-associating autotransporter
SAR	structure-activity relationship
Ser	serine
SPR	surface plasmon resonance
SRRPs	serine-rich repeat proteins
TFP	type IV pili
Thr	threonine
TNFR	tumor necrosis factor receptor
TPS	two-partner secretion
TRADD	type 1 associated DEATH domain protein
UDP	uridine diphosphate
UDP-GlcNAc	uridine diphosphate N-acetylglucosamine
UPEC	uropathogenic E. coli
VS	virtual screening
Xyl	xylose

## Footnotes

^1^ www.iith.ac.in/EK3D/
