# Corrigendum: Opportunities and Challenges of Bacterial Glycosylation for the Development of Novel Antibacterial Strategies

**DOI:** 10.3389/fmicb.2021.803203

**Published:** 2021-11-18

**Authors:** Liubov Yakovlieva, Julius A. Fülleborn, Marthe T. C. Walvoort

**Affiliations:** Faculty of Science and Engineering, Stratingh Institute for Chemistry, University of Groningen, Groningen, Netherlands

**Keywords:** pathogenic bacteria, glycosylation, antivirulence, antibacterial strategies, metabolic oligosaccharide engineering

**Error in Figure/Table**

In the original article, there was a mistake in **Figure 5** as published. **There was a mistake in the structure of CMP-KDN molecule, where at the anomeric position an OH group was drawn instead of COOH **. The corrected **Figure 5** appears below.

**Figure 5:**
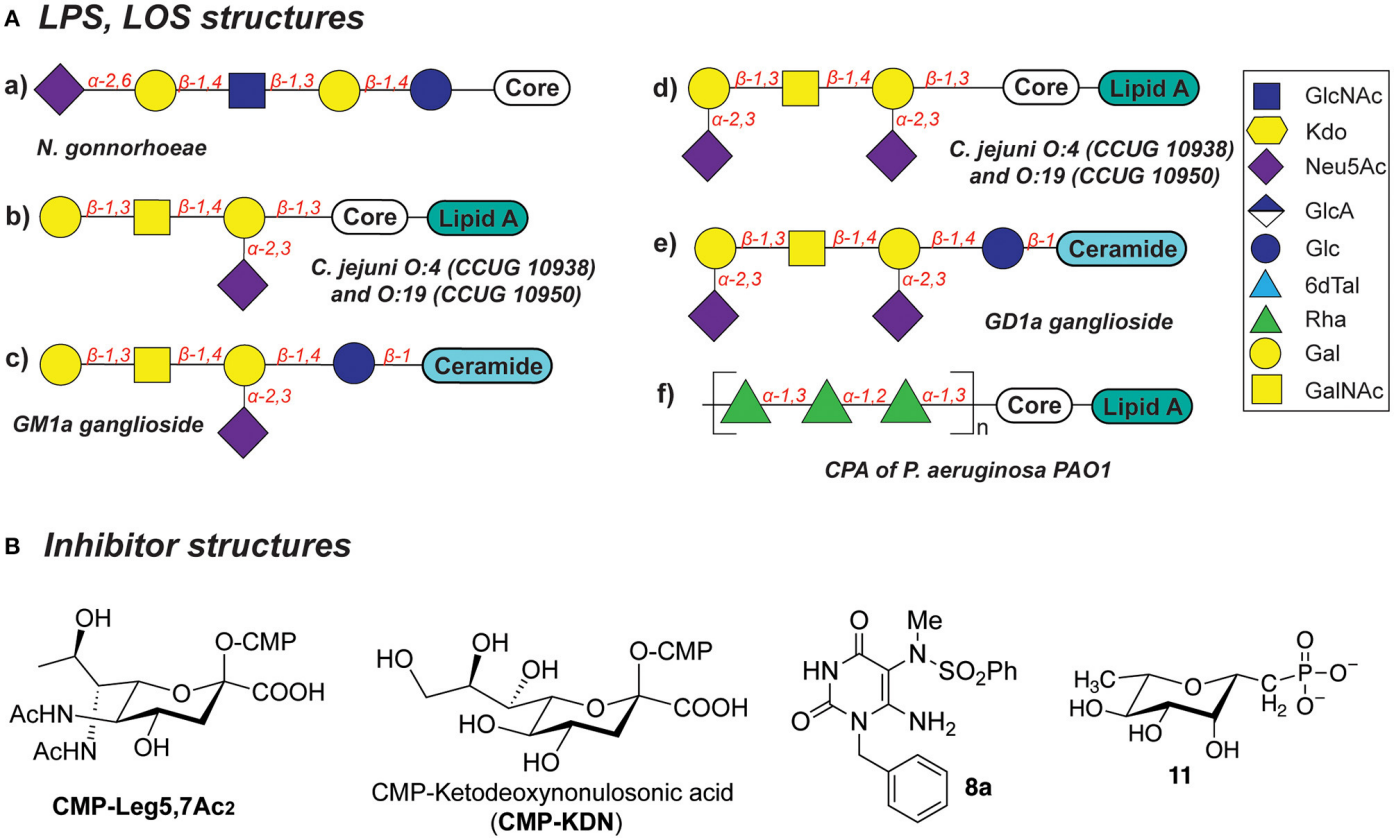
**(A)** Structures of LOS described in this section. **(B)** Structures of inhibitors discussed in this section.

The authors apologize for this error and state that this does not change the scientific conclusions of the article in any way. The original article has been updated.

## Publisher's Note

All claims expressed in this article are solely those of the authors and do not necessarily represent those of their affiliated organizations, or those of the publisher, the editors and the reviewers. Any product that may be evaluated in this article, or claim that may be made by its manufacturer, is not guaranteed or endorsed by the publisher.

